# Diurnal Variation of Intravenous Thrombolysis Rates for Acute Ischemic Stroke and Associated Quality Performance Parameters

**DOI:** 10.3389/fneur.2017.00341

**Published:** 2017-07-21

**Authors:** Björn Reuter, Tamara Sauer, Christoph Gumbinger, Ingo Bruder, Stella Preussler, Werner Hacke, Michael G. Hennerici, Peter A. Ringleb, Rolf Kern, Christian Stock, Stroke Working Group of Baden-Wuerttemberg

**Affiliations:** ^1^Department of Neurology and Geriatrics, Helios Klinik Müllheim, Müllheim, Germany; ^2^Department of Neurology and Neurophysiology, Medical Center – University of Freiburg, Freiburg, Germany; ^3^Department of Neurology, Universitätsmedizin Mannheim, Heidelberg University, Heidelberg, Germany; ^4^Department of Neurology, Heidelberg University, Heidelberg, Germany; ^5^Office for Quality Assurance in Hospitals (GeQiK), Baden-Wuerttembergische Hospital Association, Stuttgart, Germany; ^6^Institute of Medical Biometry and Informatics, Heidelberg University, Heidelberg, Germany; ^7^Department of Neurology, Klinikum Kempten, Kempten, Germany; ^8^Division of Clinical Epidemiology and Aging Research, German Cancer Research Center (DKFZ), Heidelberg, Germany

**Keywords:** ischemic stroke, daytime, thrombolysis, onset-to-door time, door-to-imaging time, door-to-needle time

## Abstract

**Introduction:**

Based on data from the Baden-Wuerttemberg stroke registry, we aimed to explore the diurnal variation of acute ischemic stroke (IS) care delivery.

**Materials and methods:**

92,530 IS patients were included, of whom 37,471 (40%) presented within an onset-to-door time ≤4.5 h. Daytime was stratified in 3-h time intervals and working vs. non-working hours. Stroke onset and hospital admission time, rate of door-to-neurological examination time ≤30 min, onset-/door-to-imaging time IV thrombolysis (IVT) rates, and onset-/door-to-needle time were determined. Multivariable regression models were used stratified by stroke onset and hospital admission time to assess the relationship between IVT rates, quality performance parameters, and daytime. The time interval 0:00 h to 3:00 h and working hours, respectively, were taken as reference.

**Results:**

The IVT rate of the whole study population was strongly associated with the sleep–wake cycle. In patients presenting within the 4.5-h time window and potentially eligible for IVT stratification by hospital admission time identified two time intervals with lower IVT rates. First, between 3:01 h and 6:00 h (IVT rate 18%) and likely attributed to in-hospital delays with the lowest diurnal rate of door-to-neurological examination time ≤30 min and the longest door-to-needle time Second, between 6:01 h and 15:00 h (IVT rate 23–25%) compared to the late afternoon and evening hours (IVT rate 27–29%) due to a longer onset-to-imaging time and door-to-imaging time. No evidence for a compromised stroke service during non-working hours was observed.

**Conclusion:**

The analysis provides evidence that acute IS care is subject to diurnal variation which may affect stroke outcome. An optimization of IS care aiming at constantly high IVT rates over the course of the day therefore appears desirable.

## Introduction

Despite current advances in vascular recanalization strategies, the key factor for successful acute ischemic stroke (IS) treatment is time (1, 2). This comprises early admittance by emergency medical services (EMS) to a hospital with sufficient stroke expertise, immediate neurological examination, transfer to brain imaging, and final decision-making whether the patient is suitable for IV thrombolysis (IVT) and/or endovascular therapy or not. Widely used indicators to measure the efficiency of stroke care are the onset-to-imaging time (OIT), door-to-imaging time (DIT), and the door-to-needle time (DNT) for those eligible to receive IVT (3, 4). In clinical routine several factors might contribute to pre- and in-hospital delays in diagnosis and treatment. These can be classified to be of either organizational nature (misinterpretation of stroke symptoms by patients and/or EMS, failure to pre-notify the hospital staff, missing or insufficient standard operation procedures) or to be patient-related (stroke severity, need for antihypertensive treatment, vomiting, mechanical ventilation, or hindered decision-making due to relevant comorbidities and/or premedication) (5–11). Studies from England, Australia, and the Safe Implementation of Treatments in Stroke-International Stroke Thrombolysis Register (SITS-ISTR) reported a less-efficient provision of stroke care during the night hours and non-working hours (11–13). Moreover, the general risk to suffer an IS is known to display a circadian rhythm and was observed to be highest in the morning hours (14, 15). When it is taken into account that most patients with wake-up strokes are additionally admitted in the morning hours, the aim to ensure a constantly high quality of stroke care 24/7 might be ambitious not only off-time but also during regular working-hours (16). Based on data from the Baden-Wuerttemberg stroke registry, we explored diurnal variation in IVT rates and associated quality performance parameters in patients with acute IS.

## Materials and Methods

We performed a retrospective analysis of patients hospitalized with acute IS registered in a stroke registry in the federal state of Baden-Württemberg, Germany. The analysis covers pre- and in-hospital care and is stratified by both stroke onset time and hospital admission time. The study was approved by the ethics committee of the Medical Faculty, University of Heidelberg (S339-2012) and by the governing board of the office for quality assurance in hospitals (Geschäftsstelle für Qualitätssicherung im Krankenhaus, GeQiK).

### Setting, Eligibility Criteria, and Study Size

A comprehensive description of the Baden-Württemberg stroke registry is provided elsewhere (17). In brief, since 2004 all stroke patients hospitalized within 7 days after stroke onset and at minimum 18 years of age are registered. Pseudonymized data are transferred to the Office for Quality Assurance in Hospitals. Data covering a period of 5 years, from January 1, 2008, to December 31, 2012, were analyzed. 108,933 patients were identified of having suffered an IS according to the International Classification of Diseases-10 discharge diagnosis I63, of whom 92,530 patients were included in the analysis. Exclusion criteria were admission for either diagnosis or therapy only (*N* = 4,592), secondary transfer from another hospital (*N* = 7,700), in-hospital strokes (*N* = 2,992), and endovascular treatment (*N* = 1,119).

### Variables

The following variables were used from electronic patient records: year of admission, patient demographic data, medical history on diabetes mellitus and previous cerebrovascular events, stroke onset time, hospital admission time, door-to-neurological examination time ≤30 min, OIT, DIT, IVT including onset-to-needle time (ONT) and DNT, and level of hospital care. Stroke severity at admission was assessed using the National Institutes of Health Stroke Scale (NIHSS) score and the modified Rankin Scale (mRS) score. An estimated pre-stroke mRS score was recorded at admission to document acute deterioration of functional ability.

The exact time of stroke onset was unknown for 53% of the whole study population (*N* = 48,673) and 28% of the subgroup of patients presenting within the 4.5-h time window (*N* = 10,372). For these patients with unwitnessed stroke the following imputation strategy was used: stroke onset was categorized into time intervals based on the time when the patient was last seen normal (in 2008: <3, 3 to <6, 6 to <24, and ≥24 h before admission; from 2009 to 2012: ≤2, >2 to 3, >3 to 6, >6 to 24, >24 to 48, and >48 h before admission). A random stroke onset time was then assigned assuming a uniform distribution over the respective interval.

### Statistical Analysis

Patient characteristics were analyzed descriptively for the whole study population and for the subgroup of patients presenting within the 4.5-h time window. Furthermore, the time of stroke onset, hospital admission time, IVT rate, OIT, DIT, ONT, and DNT were explored descriptively across the day. The daytime was stratified in 3-h time intervals (0:00 h to 3:00 h, 3:01 h to 6:00 h, …, 21:01 h to 23:59 h) and working vs. non-working hours (8:01 h to 17:00 h vs. 17:01 h to 8:00 h). Stratification by weekday and weekend was not performed in the analysis. Multivariable regression models were used to assess potential associations between stroke onset time or hospital admission time and time-based quality performance parameters. The time interval 0:00 h to 3:00 h and working hours were taken as reference, respectively. Logistic regression was used to model binary outcomes and linear models of the log(time) were used for variables measuring duration. The models were adjusted for the year of admission, stroke service level of the admitting hospital, and the following patient characteristics: age, sex, comorbidities, pre-stroke mRS score, and NIHSS score at admission. Patients with missing data on the response or explanatory variables (other than stroke onset time) were excluded from the respective analyses. All statistical tests were two-sided. Since no adjustment for multiplicity was performed, the *p*-values need to be interpreted only descriptively and do not allow confirmatory statements. The analyses were carried out using SAS 9.4 (SAS Institute Inc., Cary, NC, USA).

## Results

### Study Population

Of the whole study population *N* = 37,414 (40%) were admitted within 4.5 h after stroke onset (Table 1). The median age was 76 years and the gender ratio was balanced. Prior to stroke 61% of the whole study population were free of any functional disability (mRS score 0).

**Table 1:** Study population.

Variable	
Patients, *N*	92,530
Patients admitted within the 4.5-h time window, *N* (%)	37,414 (40)
Year of admission, *N* (%)	
2008	16,801 (18)
2009	17,856 (19)
2010	18,979 (21)
2011	19,229 (21)
2012	19,674 (21)
Age, median (IQR)	76 (68, 83)
Male sex, *N* (%)	46,537 (50)
Estimated pre-stroke mRS score, *N* (%)	
0	56,408 (61)
1	12,334 (13)
2	11,195 (12)
3	8,181 (9)
4	3,535 (4)
5	877 (1)
NIHSS score at admission, median (IQR)	4 (2, 9)
Comorbidities, *N* (%)	
Atrial fibrillation	27,234 (29)
Diabetes mellitus	25,969 (28)
Prior stroke event	24,234 (26)
Admitting hospital, *N* (%)	
Stroke center	21,268 (23)
Regional SU	17,260 (19)
Local SU	36,443 (39)
Hospitals w/o SU	17,559 (19)

*IQR, interquartile range; mRS, modified Rankin scale; NIHSS, National Institute of Health Stroke Scale; SU, stroke unit*.

### Time of Stroke Onset and Hospital Admission Time

**Figure 1:**
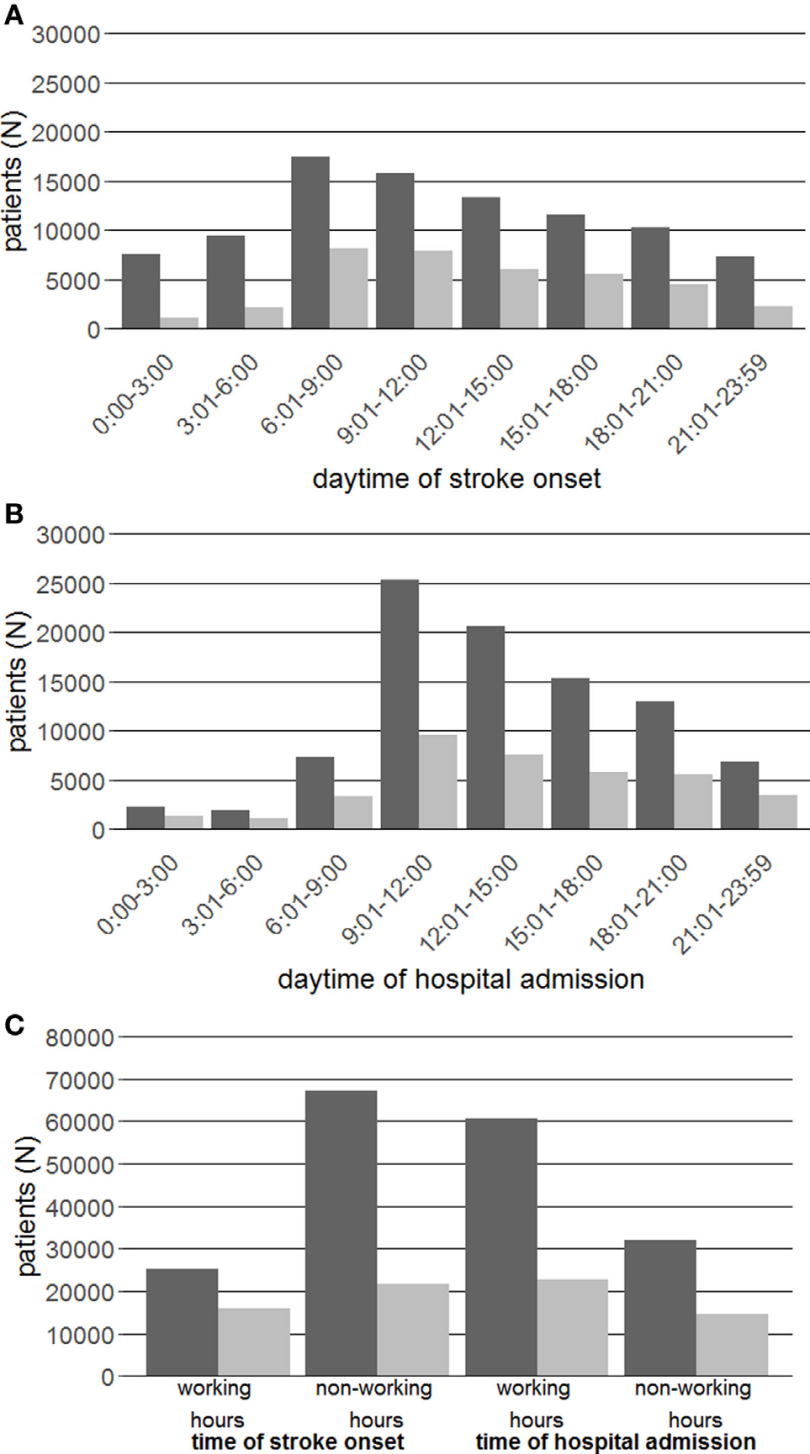
Absolute numbers of the whole study population (dark bars) and the proportion of patients admitted within the 4.5-h time window (light bars) are presented stratified by daytime of stroke onset **(A)**, daytime of hospital admission **(B)**, and onset/admission during working vs. non-working hours **(C)**.

Figure 1 shows the distribution of patients according to stroke onset time (the patients and EMS perspective) and hospital admission time (the hospitals perspective). The highest risk to suffer an IS was observed between 6:01 h and 9:00 h (19% of the whole study population), with a continuous decline over the course of the day (Figure 1A). The proportion of patients presenting within the 4.5-h time window was closely connected to the sleep–wake cycle, with a maximum of 50% between 9:01 h and 12:00 h and a minimum of 14% between 0:00 h and 3:00 h.

For the time of hospital admission a 3-h shift in the distribution compared to time of stroke onset was observed with the highest proportion being admitted between 9:01 h and 12:00 h (27% of the whole study population), again followed by a steady decline over the course of the day. Although in terms of absolute numbers most patients with onset-to-door time (ODT) ≤ 4.5 h were hospitalized during daytime hours, their respective proportion was highest during nighttime hours (maximum: 57% between 0:00 h and 3:00 h; minimum: 36% between 12:01 h and 15:00 h).

When the daytime was categorized into working and non-working hours, more than two-thirds of the ISs occurred during non-working hours (72%), of whom only 32% presented within the 4.5-h time window (Figure 1C). Of the remaining 27% suffering IS during working hours, 63% presented within the 4.5-h time window. When looking at the time of hospital admission the proportions were inverse with 65% being admitted during working hours. Of these, 38% presented within the 4.5-h time window compared to 46% during non-working hours.

### Rate of Early Neurological Examination

Of the whole study population, 75% were examined by a neurologist within 30 min after admission (minimum: 72% between 3:01 h and 6:00 h, maximum: 76% between 9:01 h and 12:00 h, Table S1 in Supplementary Material). The percentage was observed to be slightly higher in those presenting within the 4.5-h time window (minimum: 71% between 3:01 h and 6:00 h, maximum: 78% between 15:01 h and 18:00 h). After adjustment for patient and hospital characteristics the rate of door-to-neurological examination time ≤30 min in both study populations was statistically significantly higher during daytime and working hours, respectively.

### IVT Rates

**Figure 2:**
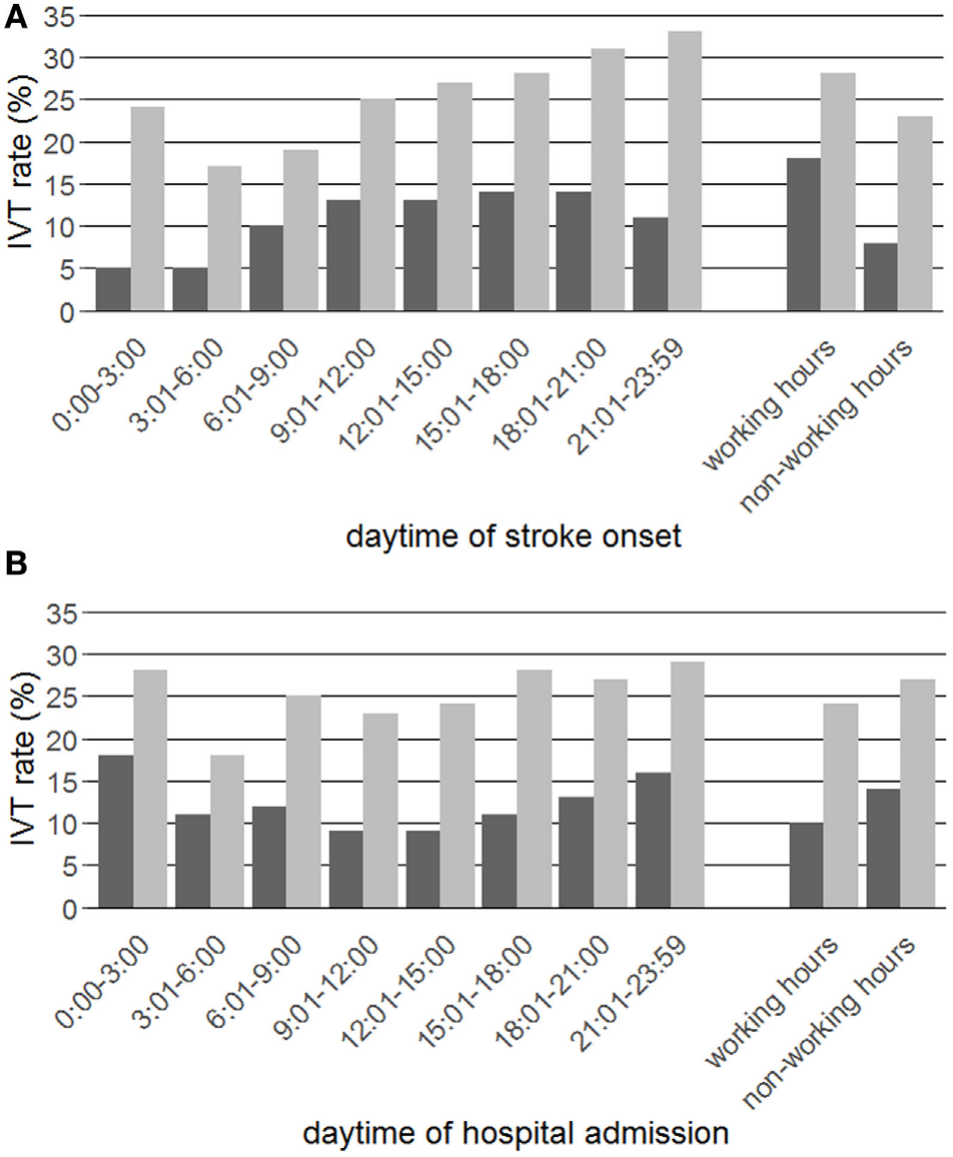
*N* = 10,104 out of 92,530 patients were treated with IV thrombolysis (IVT, dark bars), of whom *N* = 9,424 out of 37,471 were admitted within the 4.5-h time window (light bars). IVT rates are presented in percentages of the respective groups stratified by 3-h time intervals and working vs. non-working hours for the daytime of stroke onset **(A)** and hospital admission **(B)**.

When stratified by stroke onset time, the IVT rate of patients presenting within the 4.5-h time window showed a constant increase over the course of the day from 17% between 3:01 h and 6:00 h to 33% between 21:01 h and 23:59 h (Figure 2A; Table 2). The IVT rate of the whole study population increased significantly during the morning hours with a plateau of 13–14% between 09:01 h and 21:00 h and was lowest between 0:00 h and 6:00 h with 5%. IVT rates during non-working hours were generally observed to be lower compared to working hours (23 vs. 28% for the subgroup presenting within the 4.5-h time window; 8 vs. 18% for the whole study population).

**Table 2:** Probability of IV thrombolysis (IVT) stratified by stroke onset and hospital admission time (binary logistic regression analysis).

Variable	Whole study population			Patients admitted within the 4.5-h time window		
	IVT rate	Adjusted OR (95%-CI)	*P*-value	IVT rate	Adjusted OR (95%-CI)	*P*-value
	*N* (%)			*N* (%)		
**Stroke onset time**						
0–3 h	345 (4)	1.0 (ref.)		236 (26)	1.0 (ref.)	
>3–6 h	467(6)	1.12 (0.96–1.31)	0.13	355 (19)	0.73 (0.59–0.89)	<0.01
>6–9 h	1,634 (11)	2.45 (2.16–2.77)	<0.001	1,523 (21)	0.99 (0.83–1.18)	0.92
>9–12 h	2,048 (15)	3.54 (3.13–4.01)	<0.001	1,975 (28)	1.44 (1.21–1.71)	<0.001
>12–15 h	1,697 (15)	3.51 (3.09–3.98)	<0.001	1,619 (30)	1.63 (1.37–1.94)	<0.001
>15–18 h	1,570 (16)	3.59 (3.16–3.08)	<0.001	1,503 (30)	1.58 (1.32–1.88)	<0.001
>18–21 h	1,402 (16)	3.54 (3.11–4.02)	<0.001	1,345 (34)	1.78 (1.49–2.12)	<0.001
>21–23:59 h	760 (12)	2.49 (2.17–2.86)	<0.001	711 (36)	1.87 (1.55–2.27)	<0.001
Working hours	4,483 (20)	1.0 (ref.)		4,400 (31)	1.0 (ref.)	
Non-working hours	5,440 (10)	0.37 (0.36, 0.39)	<0.001	4,867 (26)	0.74 (0.70–0.78)	<0.001
**Hospital admission time**						
0–3 h	397 (20)	1.0 (ref.)		351 (30)	1.0 (ref.)	
>3–6 h	207(12)	0.48 (0.40–0.59)	<0.001	177 (20)	0.55 (0.44–0.69)	<0.001
>6–9 h	884(14)	0.66 (0.57–0.76)	<0.001	811 (27)	0.94 (0.80–1.10)	0.44
>9–12 h	2,271 (11)	0.56 (0.49–0.63)	<0.001	2,118 (25)	0.94 (0.81–1.09)	0.40
>12–15 h	1,851 (11)	0.59 (0.52–0.67)	<0.001	1,757 (26)	1.03 (0.88–1.19)	0.73
>15–18 h	1,652 (13)	0.72 (0.63–0.82)	<0.001	1,567 (31)	1.25 (1.07–1.45)	<0.01
>18–21 h	1,581 (14)	0.77 (0.69–0.90)	<0.001	1,495 (30)	1.17 (1.00–1.36)	0.05
>21–23:59 h	1,080 (18)	1.01 (0.88–1.16)	0.91	991 (32)	1.26 (1.07–1.48)	<0.01
Working hours	5,703 (11)	1.0 (ref.)		5,373 (27)	1.0 (ref.)	
Non-working hours	4,220 (15)	1.34 (1.28–1.40)	<0.001	3,894 (30)	1.08 (1.02–1.14)	<0.001

*OR estimates are adjusted for age, sex, pre-stroke mRS scores, National Institute of Health Stroke Scale score at admission, prior stroke event, diabetes, atrial fibrillation, admitting facility, and ward. Numbers do not add up to group totals in Table 1 due to missing values for explanatory variables (*N* = 13,781 for the whole study population and *N* = 4,275 for the subgroup admitted ≤4.5 h after stroke onset). Therefore, percentages are not equal to Figure 2*.

*CI, confidence interval; mRS, modified Rankin scale; OR, odds ratio*.

When stratified by hospital admission time, the diurnal IVT rates of patients presenting within the 4.5-h time window demonstrated a single dip between 3:01 h and 6:00 h (18%) and, less pronounced compared to stratification by stroke onset time, a moderate increase over the course of the day from 23 to 25% between 6:01 h and 12:00 h up to 29% between 21:01 h and 23:59 h (Figure 2B; Table 2). The IVT rates of the whole study population were highest during the nighttime hours between 0:00 h and 3:00 h (18%) and lowest during the daytime hours between 9:01 h and 12:00 h (9%). During non-working hours, IVT rates were higher compared to working hours (27 vs. 24% for patients presenting within the 4.5-h time window; 14 vs. 10% for the whole study population).

### Time-Based Stroke Quality Performance Parameters

**Figure 3:**
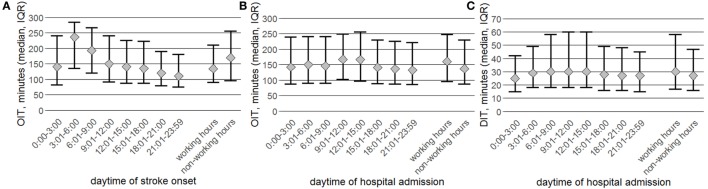
Data are stratified by 3-h time intervals and working vs. non-working hours for patients admitted within 4.5 h after stroke onset (*N* = 37,471). Presented is the onset-to-imaging time (OIT, median and interquartile range) stratified by stroke onset time **(A)** and hospital admission time **(B)** and the door-to-imaging time (DIT, median and interquartile range) stratified by hospital admission time **(C)**.

In the subgroup presenting within the 4.5-h time window the median OIT stratified by stroke onset time was longest in the early morning hours, most likely attributed to wake-up stroke patients, and then steadily declined over the course of the day (Figure 3A; Table S2 in Supplementary Material). The median OIT of the whole study population was observed to be associated with the sleep–wake cycle and shortest during daytime hours between 6:01 h and 18:00 h (Table S2 in Supplementary Material).

When stratified by hospital admission time the median OIT of patients presenting within the 4.5-h time window was observed to be more stable with a significant delay of approximately 20 min between 9:01 h and 15:00 h (Figure 3B; Table S2 in Supplementary Material). For the whole study population the longest OIT was observed during daytime hours (Table S3 in Supplementary Material). The median DIT of both study populations was statistically significantly shorter during the nighttime/non-working hours compared to daytime/working hours (Figure 3C; Table S4 in Supplementary Material).

**Figure 4:**
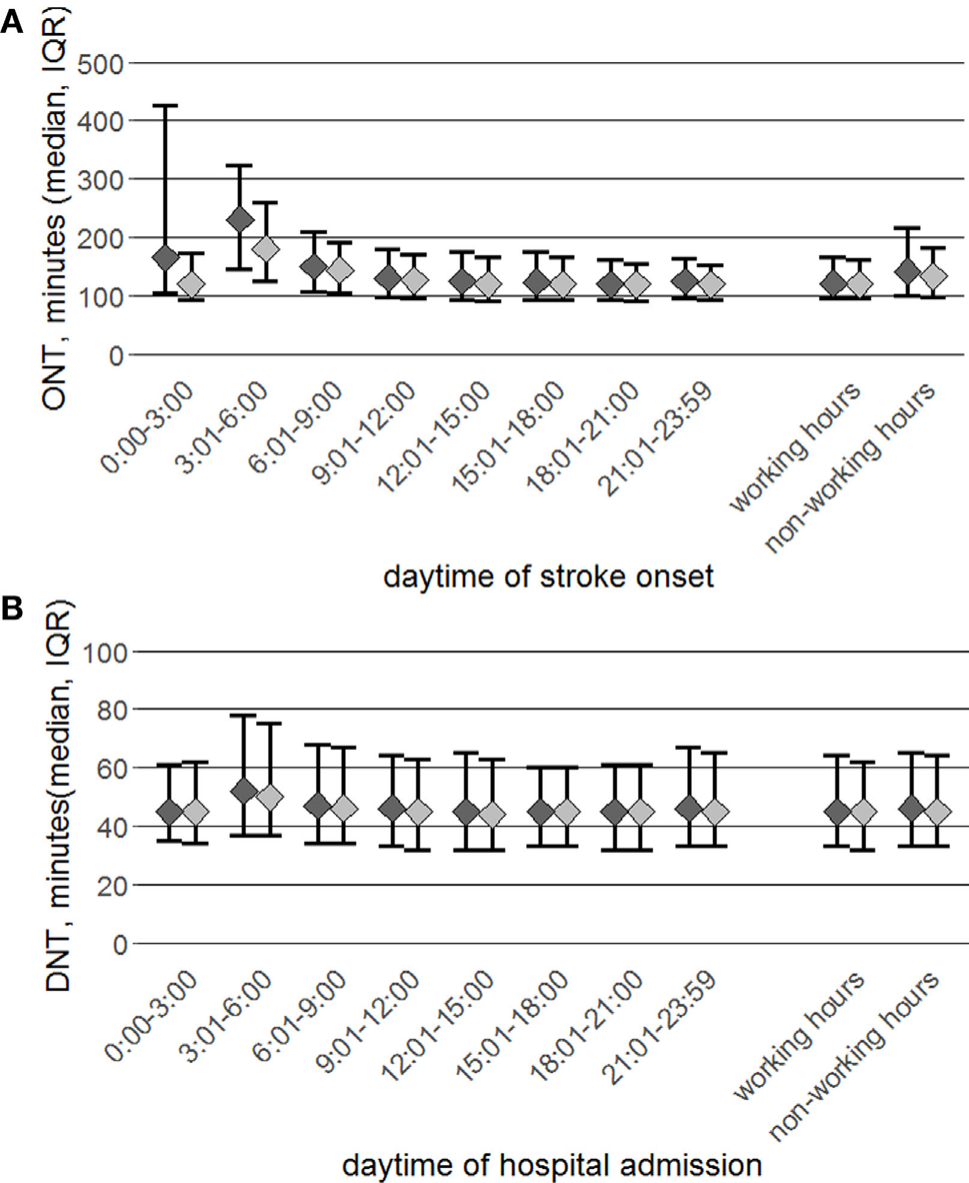
Data are stratified by 3-h time intervals and working vs. non-working hours for group totals (*N* = 10,104, dark dots) and patients admitted within the 4.5-h time window (*N* = 9,424, light dots). Presented is the onset-to-needle time (ONT), median, and interquartile range **(A)** and door-to-needle time (DNT) **(B)**, for both groups.

Between 9:01 h and 3:00 h (patients presenting within the 4.5-h time window) and 9:01 h and 23:59 h (whole study population), the median ONT for patients receiving IVT was constantly between 120 and 130 min and only marginally shorter for the subgroup with ODT ≤4.5 h (stratification by stroke onset time, Figure 4A; Table S2 in Supplementary Material). In both study groups, the median ONT increased remarkably during the night and early morning hours. Correspondingly, during non-working hours a significant delay compared to working-hours was observed (patients presenting within the 4.5-h time window 13 min, whole study population 21 min, *p* < 0.001 each). A similar, but less pronounced pattern appeared for the ONT stratified by hospital admission time (data not shown).

When stratified by hospital admission time the median DNT of both study groups was almost equal and stable over the course of the day except for a delay in the early morning hours between 3:01 h and 6:00 h (Figure 4B; Table S4 in Supplementary Material).

## Discussion

This study explored the interrelations between stroke onset time, hospital admission time, IVT rates and time-based quality performance parameters from 92,530 IS patients collected in a large stroke registry in Germany. The analysis of a whole IS study population and the subgroup presenting within the 4.5-h time window stratified by both stroke onset time and hospital admission time allows the precise identification and interpretation of diurnal variation in IVT rates. This analytical approach facilitates the evaluation of stroke care delivery and is preferable to investigating one of the two groups and either stroke onset or hospital admission time alone. Diurnal variation of process quality parameters needs to be interpreted in the context of various factors. These are the daytime of IS onset, hospital admission time, the absolute number of IS patients being admitted in the chosen time interval and the respective proportion of patients being admitted within a time frame enabling the hospital to offer IVT.

Our analysis reconfirms a very distinct diurnal pattern of IS onset. The majority of IS occurred over the daytime hours with a strong peak in the morning hours 6:01 h to 9:00 h, as was previously observed (14, 18–20). The proportion of patients admitted within the 4.5-h time window was observed to be lowest when IS occurred during nighttime hours 0:00 h to 6:00 h and highest during daytime and early evening hours 6:01 h to 21:00 h and thus confirms a direct relationship with the sleep–wake cycle (20). Hospital admission rates increased remarkably between 9:01 h and 12:00 h with the wake-up stroke patients from the night being additionally admitted and then showed a constant decline over course of the day. Admission rates during the nighttime hours were observed to be rather marginal, as shown previously (21). Stratification by hospital admission time revealed an inverse picture: the proportion of IS patients admitted within the 4.5-h time window was highest during the evening and nighttime hours 21:01 h to 6:00 h and lowest during the daytime hours 9:01 h to 18:00 h. This result might be best explained by delayed admission of wake-up stroke patients and patients primarily attending general practitioners during daytime hours (22, 23). Finally, less than one-third of the ISs occurred during working hours, but approximately two-thirds were admitted in this time interval.

### Diurnal IVT Rates and Associated Quality Performance Parameters—General Findings

The IVT rates and associated quality performance parameters of both study groups demonstrated a very different pattern, depending on stratification. When the IVT rate of the whole study population was stratified by stroke onset time a strong association with the sleep–wake cycle became evident, with a much higher IVT rate during daytime and early evening hours compared to the late evening and night hours. IS onset during daytime is associated with early hospital arrival (24). Indeed, this observation corresponded well with the respective proportion of patients presenting within the 4.5-h time window and is further supported by a significantly shorter OIT during the daytime and early evening hours. A statistically significantly delayed ONT was observed only for the night hours between 0:00 h and 6:00 h compared to the other time intervals, thus representing patients treated off-label beyond the 4.5-h time window.

Stratification by hospital admission time of the whole study population’s IVT rate revealed an inverse pattern compared to the stroke onset time, with the highest IVT rate observed between 21:01 h and 3:00 h, which on the contrary was lowest between 9:01 h and 15:00 h. This seems plausible since nighttime admission rates were low but with a high proportion of patients presenting within the 4.5-h time window and is further in line with a shorter OIT during nighttime hours. Our observation might be best explained by the assumption that IS patients with stroke onset in the evening and early night hours and seeking for immediate medical attention have no other choice than to call EMS with subsequent early hospitalization (25, 26).

### Intervals with Impaired Stroke Care Delivery for Patients Potentially Eligible for IVT

Within the subgroup of patients presenting within the 4.5-h time window, we identified two time intervals with suspected reduced quality of stroke care. First, stratification by hospital admission time demonstrated a noticeable dip of the IVT rate in the early morning hours 3:01 h to 6:00 h. In this time interval, only *N* = 1,015 patients were admitted to approx. 140 hospitals over a 5-year period, representing 3% of the study group. The low IVT rate seems to be attributed to delays of in-hospital stroke care delivery, since the pre-hospital transportation time was not observed to be delayed. Indeed, a consistent (however, due to the low admission numbers in this time interval statistically non-significant) deterioration of in-hospital performance parameters was recognized, with the lowest proportion of patients being neurologically examined within 30 min after admission and the longest DNT with additional 5 min compared to daytime levels. We believe that this observation might reflect a structural shortcoming of in-hospital stroke care between 3:01 h and 6:00 h, as for instance overnight absence or on-call service of neurologists and/or radiologists. Except for the time interval 3:01 h to 6:00 h, the diurnal DNT was observed to be quite stable and close to the <40 min aim for 50% of the patients as defined in the SITS Watch project (27).

Second, when stratified by stroke onset time the subgroup of patients within the 4.5-h time window revealed an increasing IVT rate over the course of the day which almost doubled from 18% between 3:01 h and 6:00 h to 33% between 21:01 h and 23:59 h and corresponded with a respectively shorter OIT. A similar, however less pronounced, pattern was also observed after stratification by hospital admission time. This seems to be a consequence of the diurnal stroke incidence with a subsequently high overall hospital admission rate between 6:01 h and 15:00 h, thereby delaying a time-efficient pre- and in-hospital stroke service for IS patients within the 4.5-h time window. Although the OIT of the whole study population stratified by stroke onset time does not indicate a prolonged EMS transportation time between 9:01 h and 15:00 h, the median OIT of those patients within a treatable time window decreased continuously from 150 min between 9:01 h and 12:00 h to 110 min between 21:01 h and 23:59 h. Possible explanations for this finding are that (A) during daytime hours a relevant proportion of acute stroke patients might have primarily attended a general physician which subsequently delays adequate diagnostics and treatment, and (B) that EMS are overburdened in the morning and early afternoon hours by the numbers of acute stroke patients and patients with cognate diseases seeking medical attention (22, 23, 28). As a consequence of the overall high stroke admission rate during daytime hours the DIT increased slightly between 9:01 h and 15:00 h and much less compared to the whole study group.

Possible strategies to overcome pre- and in-hospital bottlenecks might comprise diurnal adjustment of EMS transportation capacities and brain imaging capacities being exclusively assigned to emergency care in the morning and early afternoon hours. Furthermore, patients primarily contacting their general practitioner should be immediately advised to call EMS service (25). This might be achieved during non-working hours by detailed advice on answering machines and during working-hours by standardized stroke symptom assessment tools and training for receptionists and doctors, so that any kind of appointment or home visit is avoided (22, 23).

### Stroke Service during Non-Working Hours

Several studies from the UK, Australia, and SITS-ISTR, but not studies from Germany, France and South Korea reported in-hospital delays in processes of care during non-working hours (11–13, 29–33). In our analysis the IVT rate during non-working hours was observed to be higher than during working-hours. This was the case even though the probability of early neurological examination within 30 min after admission was lower during non-working hours, most likely because not all hospitals provide a 24/7 neurological presence in the emergency department. However, the DIT was significantly shorter during non-working hours, possibly due to the generally lower admission rate during non-working hours, while the DNT was equal in working and non-working hours. In summary, except for the time period 3:01 h to 6:00 h our data provide no evidence for a compromised in-hospital stroke service during non-working hours.

As a general limitation of stroke registry analyses, unlikely data from carefully and prospectively planned clinical studies, the source data could not be checked as a whole on accuracy. However, the office for quality assurance in hospitals performs predefined logical and range checks (plausibility checks) of the source data and annual reports on data quality are provided for all participating hospitals (17). Second, the exact time of stroke onset was partially unknown, as is common in routine stroke care. For these patients an imputation strategy (based on known and reasonably narrow time interval of stroke onset) was deemed sensible in order to perform calculations on exact time data and was considered unlikely to severely bias results. Third, it is well conceivable that the acute stroke care delivery is subject to considerable clinical heterogeneity across the large number of hospitals, e.g., it may vary by the level of stroke care which ranges from maximum possible care at university clinics to hospitals without stroke units. Investigating subgroup differences in diurnal variation therefore might be warranted as complementing evidence but was considered beyond the scope of the present analysis which in many respects “averages” over a large population of patients and also large number of hospitals.

## Conclusion

Our analysis provides evidence that IS care is subject to diurnal variation which may affect stroke outcomes. Time intervals with lower IVT rates were identified (1) between 3:01 h and 6:00 h and attributed to in-hospital delays, and (2) between 6:01 h and 15:00 h compared to the late afternoon and evening hours, due to a combination of pre- and in-hospital delays which are best explained by high overall IS admission rates. No evidence for a compromised stroke service during non-working hours was observed. The presented data might be helpful to optimize IS care aiming at constantly high levels of IVT over the course of the day.

## Availability of Data and Materials

The statistical code is available from the corresponding author.

## Author Contributions

BR, TS, CG, RK, and CS designed the study. BR analyzed data and wrote the paper. CS designed and performed the statistical analysis, contributed to draft versions of the paper, and revised the paper. CG, TS, HW, CD, PR, WH, and MH were involved in planning of analysis and in interpretation of data. SP was involved in the statistical analysis and revised the paper. IB was involved in preparation of source data, and RK supervised the research and revised the paper. The Stroke Working Group of the federal state of Baden-Wuerttemberg was involved in interpretation of data and revised the paper. All authors agreed to the final version of the manuscript.

## Conflict of Interest Statement

All authors declare: no support from any organization for the submitted work. RK has received speaker’s honoraria from Boehringer Ingelheim, Bayer, Pfizer/BMS, Novartis, and Daiichi Sankyo that where unrelated to this study. PR has received lecture fees and travel compensation from Boehringer-Ingelheim, Ferrer, Paion, Bayer, and Sanofi that where unrelated to this study. WH reported honoraria from Johnson & Johnson, Bayer, and advisory board fees from Boehringer Ingelheim that where unrelated to this study.
