# Supplementary material for: Diurnal Variation of Intravenous Thrombolysis Rates for Acute Ischemic Stroke and Associated Quality Performance Parameters
Source: Front Neurol. 2017 Jul 21;8:341. doi: 10.3389/fneur.2017.00341 (PMC5519519; doi:10.3389/fneur.2017.00341)
Supplement: Supplementary file 1 [file Table_1.DOCX]

| **Table S1** Rate of door-to-neurological examination time ≤30 min stratified by hospital admission time (binary logistic regression analysis) | | | | | | | |
| --- | --- | --- | --- | --- | --- | --- | --- |
|  | **Whole study population** | | |  | **Patients admitted within the 4.5h time window** | | |
| **Variable** | **Neuro exam** | **Adjusted OR  (95%-CI)** | **P value** |  | **Neuro exam** | **Adjusted OR  (95%-CI)** | **P value** |
|  | **N (%)** |  |  |  | **N (%)** |  |  |
| 0-3h | 1436 (76) | 1.0 (ref.) |  |  | 882 (78) | 1.0 (ref.) |  |
| >3-6h | 1149 (72) | 0.94 (0.79-1.12) | 0.49 |  | 609 (71) | 0.86 (0.68-1.09) | 0.21 |
| >6-9h | 4468 (74) | 1.21 (1.06-1.39) | 0.005 |  | 2100 (75) | 1.16 (0.96-1.40) | 0.13 |
| >9-12h | 15615 (76) | 1.41 (1.15-1.60) | <0.001 |  | 6241 (78) | 1.38 (1.16-1.64) | <0.001 |
| >12-15h | 12534 (75) | 1.32 (1.16-1.49) | <0.001 |  | 4970 (78) | 1.28 (1.07-1.52) | <0.01 |
| >15-18h | 9155 (74) | 1.19 (1.05-1.36) | 0.007 |  | 3786 (78) | 1.24 (1.04-1.48) | 0.02 |
| >18-21h | 7722 (74) | 1.11 (0.97-1.26) | 0.12 |  | 3621 (77) | 1.12 (0.94-1.34) | 0.21 |
| >21-23:59h | 4111 (74) | 1.05 (0.91-1.20) | 0.51 |  | 2254 (77) | 1.03 (0.86-1.25) | 0.73 |
| working hours | 36947 (76) | 1.0 (ref.) |  |  | 14975 (78) | 1.0 (ref.) |  |
| non-working hours | 19243 (74) | 0.81 (0.78-0.84) | <0.001 |  | 9488 (77) | 0.80 (0.75-0.85) | <0.001 |
| OR estimates are adjusted for age, sex, pre-stroke mRS scores, NIHSS score at admission, prior stroke event, diabetes, atrial fibrillation, admitting facility and ward. Numbers do not add up to group totals in Table 1 due to missing values for the response or explanatory variables (N=13781 for the whole study population and N=4275 for the subgroup of patients admitted ≤4.5h after stroke onset). Abbreviations: SO, stroke onset; Neuro exam, Neurological examination; CI, confidence interval; OR, odds ratio. | | | | | | | |
