# Supplementary material for: Diurnal Variation of Intravenous Thrombolysis Rates for Acute Ischemic Stroke and Associated Quality Performance Parameters
Source: Front Neurol. 2017 Jul 21;8:341. doi: 10.3389/fneur.2017.00341 (PMC5519519; doi:10.3389/fneur.2017.00341)
Supplement: Supplementary file 2 [file Table_2.DOCX]

| **Supplemental Table S2** Onset-to-imaging time stratified by stroke onset and hospital admission time (multivariable logistic regression analysis) | | | | | | | | | | | | |
| --- | --- | --- | --- | --- | --- | --- | --- | --- | --- | --- | --- | --- |
| **Variable** | | **Whole study population** | | | | |  | **Patients admitted within the 4.5h time window** | | | | |
|  |  | **minutes, median (IQR)** | **β** | **SE** | **Wald χ2** | **P value** |  | **minutes, median (IQR)** | **β** | **SE** | **Wald χ2** | **P value** |
| **Onset-to-imaging time stratified by stroke onset time** | | | | | | | | | | | | |
|  | 0-3h | 748 (487, 2031) | ref. | | | |  | 140 (82, 240) | ref. | | | |
|  | >3-6h | 510 (330, 1608) | -0.27 | 0.02 | 163.60 | <0.001 |  | 236 (135, 285) | 0.34 | 0.03 | 120.33 | <0.001 |
|  | >6-9h | 360 (199, 906) | -0.56 | 0.02 | 871.14 | <0.001 |  | 193 (120, 266) | 0.24 | 0.03 | 80.18 | <0.001 |
|  | >9-12h | 333 (150, 1490) | -0.57 | 0.02 | 886.35 | <0.001 |  | 150 (92, 240) | 0.08 | 0.03 | 8.36 | <0.01 |
|  | >12-15h | 384 (151, 1536) | -0.45 | 0.02 | 523.71 | <0.001 |  | 140 (88, 225) | 0.01 | 0.03 | 0.21 | 0.65 |
|  | >15-18h | 364 (139, 1469) | -0.43 | 0.02 | 445.80 | <0.001 |  | 135 (87, 223) | 0.02 | 0.03 | 0.71 | 0.40 |
|  | >18-21h | 853 (134, 1428) | -0.34 | 0.02 | 269.65 | <0.001 |  | 120 (79, 190) | -0.05 | 0.03 | 3.61 | 0.06 |
|  | >21-23:59h | 831 (225, 2070) | -0.15 | 0.02 | 45.76 | <0.001 |  | 110 (75, 180) | -0.07 | 0.03 | 4.53 | 0.03 |
|  | working hours | 220 (111, 640) | ref. | | | |  | 134 (90, 210) | ref. | | | |
|  | non-working hours | 611 (262, 1660) | 0.64 | 0.01 | 4120.36 | <0.001 |  | 170 (70, 256) | 0.14 | 0.01 | 250.08 | <0.001 |
| **Onset-to-imaging time stratified by hospital admission time** | | | | | | | | | | | | |
|  | 0-3h | 275 (124, 740) | ref. | | | |  | 142 (88, 239) | ref. | | | |
|  | >3-6h | 313 (140, 633) | -0.02 | 0.04 | 0.15 | 0.69 |  | 150 (90, 240) | -0.02 | 0.03 | 0.21 | 0.65 |
|  | >6-9h | 403 (159, 1060) | 0.20 | 0.03 | 39.83 | <0.001 |  | 146 (90, 240) | 0.01 | 0.03 | 0.05 | 0.82 |
|  | >9-12h | 548 (219, 1595) | 0.46 | 0.03 | 243.01 | <0.001 |  | 167 (102, 249) | 0.07 | 0.02 | 7.45 | <0.01 |
|  | >12-15h | 519 (230, 1723) | 0.46 | 0.03 | 241.12 | <0.001 |  | 166 (97, 255) | 0.05 | 0.02 | 4.10 | 0.04 |
|  | >15-18h | 495 (188, 1602) | 0.40 | 0.03 | 169.92 | <0.001 |  | 141 (89, 229) | -0.02 | 0.03 | 0.61 | 0.44 |
|  | >18-21h | 420 (154, 1367) | 0.26 | 0.03 | 73.62 | <0.001 |  | 136 (87, 226) | -0.04 | 0.03 | 2.73 | 0.10 |
|  | >21-23:59h | 325 (128, 1044) | 0.14 | 0.03 | 19.35 | <0.001 |  | 132 (86, 221) | -0.02 | 0.03 | 0.35 | 0.55 |
|  | working hours | 518 (213, 1616) | ref. | | | |  | 160 (95, 247) | ref. | | | |
|  | non-working hours | 377 (150, 1200) | -0.23 | 0.01 | 573.69 | <0.001 |  | 137 (88, 229) | -0.06 | 0.01 | 50.54 | <0.001 |
| Results are based on linear regression models of log(onset-to-imaging time) adjusted for potential confounders. Numbers do not add up to group totals presented in Table 1 due to missing values for explanatory variables (N=21248 out of 92530 for the whole cohort and N=6892 out of 37414 for the subgroup of patients admitted ≤4.5h after stroke onset). Abbrevations: IQR, interquartile range; SE, standard error. | | | | | | | | | | | | |
