# Supplementary material for: Diurnal Variation of Intravenous Thrombolysis Rates for Acute Ischemic Stroke and Associated Quality Performance Parameters
Source: Front Neurol. 2017 Jul 21;8:341. doi: 10.3389/fneur.2017.00341 (PMC5519519; doi:10.3389/fneur.2017.00341)
Supplement: Supplementary file 3 [file Table_3.DOCX]

| **Supplemental Table S3** Onset-to-needle time stratified by stroke onset time (multivariable logistic regression analysis) | | | | | | | | | | | | |
| --- | --- | --- | --- | --- | --- | --- | --- | --- | --- | --- | --- | --- |
| **Variable** | | **Whole study population** | | | | |  | **Patients admitted within the 4.5h time window** | | | | |
|  |  | **minutes, median (IQR)** | **β** | **SE** | **Wald χ2** | **P value** |  | **minutes, median (IQR)** | **β** | **SE** | **Wald χ2** | **P value** |
| **Onset-to-needle time** | |  |  |  |  |  |  |  |  |  |  |  |
|  | 0-3h | 165 (105, 426) | ref. | | | |  | 125 (94, 174) | ref. | | | |
|  | >3-6h | 230 (134, 324) | 0.001 | 0.04 | 0 | 0.98 |  | 180 (125, 259) | 0.29 | 0.04 | 63.95 | <0.001 |
|  | >6-9h | 150 (107, 210) | -0.34 | 0.03 | 99.12 | <0.001 |  | 144 (105, 192) | 0.08 | 0.03 | 7.28 | <0.01 |
|  | >9-12h | 130 (98, 180) | -0.49 | 0.03 | 214.74 | <0.001 |  | 127 (95, 170) | -0.03 | 0.03 | 0.71 | 0.40 |
|  | >12-15h | 125 (94, 175) | -0.51 | 0.03 | 226.50 | <0.001 |  | 120 (91, 165) | -0.06 | 0.03 | 3.31 | 0.07 |
|  | >15-18h | 123 (94, 175) | -0.51 | 0.03 | 224.57 | <0.001 |  | 120 (92, 165) | -0.09 | 0.03 | 4.39 | 0.04 |
|  | >18-21h | 120 (94, 161) | -0-56 | 0.03 | 264.39 | <0.001 |  | 120 (90, 155) | -0.09 | 0.03 | 8.83 | <0.01 |
|  | >21-23:59h | 125 (95, 164) | -0.51 | 0.04 | 187.49 | <0.001 |  | 120 (94, 152) | -0.08 | 0.03 | 7.23 | <0.01 |
|  | working hours | 121 (95, 165) | ref. | | | |  | 120 (95, 161) | ref. | | | |
|  | non-working hours | 142 (101, 217) | 0.20 | 0.01 | 285.96 | <0.001 |  | 133 (98, 182) | 0.08 | 0.01 | 79.08 | <0.001 |
| Results are based on linear regression models of log(onset-to-needle time) adjusted for potential confounders. Numbers do not add up to group totals presented in Table 1 due to missing values for explanatory variables (N=408 out of 10104 for the whole cohort and N=316 out of 9424 for the subgroup of patients admitted ≤4.5h after stroke onset). Abbrevations: IQR, interquartile range; SE, standard error. | | | | | | | | | | | | |
