# Supplementary material for: Diurnal Variation of Intravenous Thrombolysis Rates for Acute Ischemic Stroke and Associated Quality Performance Parameters
Source: Front Neurol. 2017 Jul 21;8:341. doi: 10.3389/fneur.2017.00341 (PMC5519519; doi:10.3389/fneur.2017.00341)
Supplement: Supplementary file 4 [file Table_4.DOCX]

| **Supplemental Table S4** Door-to-imaging time and door-to-needle time stratified by hospital admission time (multivariable logistic regression analysis) | | | | | | | | | | | | |
| --- | --- | --- | --- | --- | --- | --- | --- | --- | --- | --- | --- | --- |
| **Variable** | | **Whole study population** | | | | |  | **Patients admitted within the 4.5h time window** | | | | |
|  |  | **minutes, median (IQR)** | **β** | **SE** | **Wald χ2** | **P value** |  | **minutes, median (IQR)** | **β** | **SE** | **Wald χ2** | **P value** |
| **Door-to-imaging time** | |  |  |  |  |  |  |  |  |  |  |  |
|  | 0-3h | 29 (16, 54) | ref. | | | |  | 25 (15, 42) | ref. | | | |
|  | >3-6h | 30 (19, 58) | 0.06 | 0.05 | 1.34 | 0.25 |  | 29 (18, 49) | 0.09 | 0.06 | 1.99 | 0.16 |
|  | >6-9h | 35 (21, 76) | 0.19 | 0.04 | 25.62 | <0.001 |  | 30 (18, 58) | 0.17 | 0.05 | 13.47 | <0.001 |
|  | >9-12h | 41 (22, 87) | 0.22 | 0.03 | 41.46 | <0.001 |  | 30 (18, 60) | 0.16 | 0.04 | 15.09 | <0.001 |
|  | >12-15h | 41 (22, 89) | 0.19 | 0.03 | 30.69 | <0.001 |  | 30 (18, 60) | 0.11 | 0.04 | 6.86 | 0.01 |
|  | >15-18h | 35 (20, 74) | 0.11 | 0.04 | 10.22 | <0.01 |  | 28 (16, 49) | 0.05 | 0.04 | 1.52 | 0.22 |
|  | >18-21h | 33 (20, 68) | 0.09 | 0.04 | 6.42 | 0.01 |  | 27 (16, 48) | 0.05 | 0.04 | 1.31 | 0.25 |
|  | >21-23:59h | 30 (18, 59) | 0.09 | 0.04 | 5.29 | 0.02 |  | 27 (15, 45) | 0.04 | 0.05 | 0.80 | 0.37 |
|  | working hours | 40 (22, 85) | ref. | | | |  | 30 (17, 58) | ref. | | | |
|  | non-working hours | 32 (19, 64) | -0.11 | 0.01 | 96.27 | <0.001 |  | 27 (16, 47) | -0.07 | 0.02 | 17.83 | <0.001 |
| **Door-to-needle time** | |  |  |  |  |  |  |  |  |  |  |  |
|  | 0-3h | 45 (35, 61) | ref. | | | |  | 45 (34, 62) | ref. | | | |
|  | >3-6h | 52 (37, 78) | 0.19 | 0.06 | 8.63 | <0.01 |  | 50 (37, 75) | 0.12 | 0.07 | 3.14 | 0.08 |
|  | >6-9h | 47 (34, 68) | 0.07 | 0.05 | 2.39 | 0.12 |  | 46 (34, 67) | 0.03 | 0.05 | 0.34 | 0.56 |
|  | >9-12h | 46 (33, 64) | <0.01 | 0.04 | 0 | 0.95 |  | 45 (32, 63) | -0.03 | 0.04 | 0.59 | 0.44 |
|  | >12-15h | 45 (32, 65) | -0.05 | 0.04 | 1.71 | 0.19 |  | 44 (32, 63) | -0.09 | 0.04 | 4.66 | 0.03 |
|  | >15-18h | 45 (33, 60) | -0.01 | 0.04 | 0.15 | 0.70 |  | 45 (33, 60) | -0.05 | 0.04 | 1.40 | 0.24 |
|  | >18-21h | 45 (32, 61) | -0.04 | 0.04 | 0.89 | 0.34 |  | 45 (32, 61) | -0.06 | 0.04 | 2.20 | 0.14 |
|  | >21-23:59h | 46 (33, 67) | 0.01 | 0.04 | 0.02 | 0.89 |  | 45 (33, 65) | -0.04 | 0.05 | 0.85 | 0.36 |
|  | working hours | 45 (33, 64) | ref. | | | |  | 45 (32, 62) | ref. | | | |
|  | non-working hours | 46 (33, 65) | 0.03 | 0.02 | 3.03 | 0.08 |  | 45 (33, 64) | 0.03 | 0.02 | 3.45 | 0.06 |
| Numbers do not add up to group totals presented in Table 1 due to missing values for explanatory variables (onset-to-imaging time N=21248 out of 92530 for the whole cohort and N=6892 out of 37414 for the subgroup of patients admitted ≤4.5h after stroke onset; onset-to-needle time N=408 out of 10104 for the whole cohort and N=316 out of 9424 for the subgroup of patients admitted ≤4.5h after stroke onset). Abbrevations: IQR, interquartile range; SE, standard error. | | | | | | | | | | | | |
